# Diagnostic potential of Aldo-keto reductase 1B10 in pediatric hepatic malignancies: a narrative, hypothesis-generating translational review based on adult evidence and future research directions

**DOI:** 10.3389/fmed.2026.1972682

**Published:** 2026-10-23

**Authors:** Jianhang Luo, Peng Liao, Zheng Zeng, Qiulin Deng, Meilin Hu, Lingfeng Liu, Caizhi Huang, Qiang Yin, Jun Peng, Deliang Cao, Xin Hu, Lijian Chen

**Affiliations:** 1 Hunan Engineering Research Center for Early Diagnosis and Treatment of Liver Cancer, Hunan Province Key Laboratory of Tumor Cellular & Molecular Pathology, Cancer Research Institute, Hengyang Medical School, University of South China, Hengyang, Hunan, China; 2 Department of General Surgery I, Affiliated Children’s Hospital of Xiangya School of Medicine, Central South University (Hunan Children's Hospital), Changsha, China; 3 Department of Neonatology, Affiliated Children’s Hospital of Xiangya School of Medicine, Central South University (Hunan Children's Hospital), Changsha, China; 4 The Affiliated Nanhua Hospital, Hengyang Medical School, University of South China, Hengyang, Hunan, China; 5 Department of Clinical Laboratory, The Affiliated Children’s Hospital of Xiangya School of Medicine, Central South University (Hunan Children's Hospital), Changsha, China; 6 Geological Experiment and Testing Center of Hunan Province, Changsha, China; 7 Division of Transplantation Immunology, National Research Institute for Child Health and Development, 2-10-1 Okura, Setagaya-ku, Tokyo, Japan; 8 Department of Surgery, Nagasaki University Graduate School of Biomedical Sciences, Nagasaki, Japan

**Keywords:** Aldo-keto reductase 1B10 (AKR1B10), alpha-fetoprotein (AFP), hepatoblastoma (HB), hepatocellular carcinoma (HCC), pediatric hepatic malignancies

## Abstract

**Objective:**

The difficulty in diagnosing hepatocellular carcinoma (HCC) at an early stage is a common challenge faced in the management of malignant liver tumors in both adults and children. Serum alpha-fetoprotein (AFP) is currently the most widely used screening marker; however, it lacks sufficient sensitivity for early-stage HCC and exhibits physiologically elevated levels in infants and young children, which interferes with the accurate diagnosis of hepatoblastoma (HB). Aldo-keto reductase 1B10 (AKR1B10) is a liver cancer-associated protein that has attracted significant attention in recent years. It is frequently overexpressed in adult HCC and can be secreted into the bloodstream; however, its expression and function in pediatric liver tumors have not been reported to date. This study aimed to explore the potential research value of AKR1B10 in pediatric liver malignancies and provide a theoretical basis for expanding its clinical applications.

**Methods:**

This narrative review summarizes the molecular structure, catalytic properties, tissue expression patterns, and core molecular mechanisms driving the onset and progression of HCC associated with AKR1B10, as well as its serum diagnostic efficacy and current status of clinical translation in adult HCC. Based on the molecular overlap between adult and pediatric liver tumors in the three signaling pathways—Wnt/β-catenin, lipid metabolism, and retinoic acid signaling—and using theoretical deduction and cross-disease comparative analysis, this study proposes, for the first time, a hypothesis to extend AKR1B10 research to the field of pediatric liver tumors.

**Proposed Hypotheses:**

Based on adult HCC evidence and shared molecular pathways, we hypothesize that: (1) AKR1B10 may be upregulated in HB, which could help address the diagnostic blind spots caused by physiological fluctuations in AFP levels during infancy and early childhood; (2) AKR1B10 may serve as an auxiliary serum biomarker for AFP-negative or low-expressing HB subtypes.

**Conclusion:**

To our knowledge, this narrative review is the first to propose the potential research value of AKR1B10 in pediatric liver malignancies. This study aims to provide a theoretical foundation and translational direction for future research on AKR1B10 in the pediatric field.

## 1 Introduction

Primary liver cancer ranks third among the global causes of cancer-related death. In China, the number of deaths and mortality rate from primary liver cancer rose to second place in 2024. Among primary liver cancers, hepatocellular carcinoma (HCC) is the most common, accounting for 75%–85% of cases, and intrahepatic cholangiocarcinoma is the second most common, accounting for 10%–15% of cases (1). In adults, HCC often develops against a background of chronic liver disease and cirrhosis. The incidence of this disease varies greatly across different regions and also differs between men and women (1–3).

In the pediatric population, primary malignant liver tumors are rare, with an annual incidence of approximately 1–2 per million, accounting for approximately 1% of pediatric malignancies (4, 5). The disease profiles of the two age groups differ markedly: in children, hepatoblastoma (HB) is the predominant form, accounting for approximately 80% of pediatric malignant liver tumors, with the peak incidence occurring in children under 3 years of age (5–7); pediatric hepatocellular carcinoma (pHCC) is relatively rare and is more commonly seen in adolescents (5, 8).

Although malignant liver tumors in adults and children differ significantly in etiology, pathogenesis, and pathological classification, early diagnosis plays a common, critical role in improving the prognosis for both groups. The 5-year survival rate for advanced HCC is less than 15%, whereas the 5-year survival rate after curative resection of early-stage HCC can reach 69%–86.2% (9, 10). In children, the 3-year overall survival rate for high-risk HB is only 52%–65%, whereas it exceeds 90% for standard-risk HB (7, 11). In recent years, serum alpha-fetoprotein (AFP) combined with abdominal ultrasound remains the most widely used non-invasive screening protocol (10, 12). However, numerous studies have demonstrated that serum AFP has diagnostic limitations in both adult and pediatric liver malignancies; these diagnostic challenges serve as the direct driving force behind our exploration of novel biomarkers. Aldo-keto reductase 1B10 (AKR1B10) has entered the field of liver cancer research precisely against this backdrop. A growing body of research confirms that serum AKR1B10, as a secreted protein, outperforms AFP in the diagnosis of HCC and early-stage HCC, and that the combined use of both markers can improve diagnostic accuracy (13, 14).

It is worth noting that nearly all existing research on AKR1B10 has focused on adult HCC. To date, there has been a lack of systematic investigation into the expression patterns, functional significance, and clinical value of this protein in HB and other pediatric liver malignancies. However, adult HCC and HB share potential molecular overlaps in terms of abnormal activation of the Wnt/β-catenin pathway, lipid metabolism reprogramming, and developmental differentiation regulation (6, 15–21). This review begins with a comparison of the epidemiology and current diagnostic status of malignant liver tumors in adults and children. Based on a narrative review of the molecular mechanisms of AKR1B10 and clinical evidence in adults, we propose a hypothetical framework and validation pathways for its application in pediatric liver tumor research.

### 1.1 Literature search and evidence selection

This review is a narrative, hypothesis-generating translational review, and new quantitative meta-analyses were conducted. Literature searches were conducted from July 2025 to July 2026 in PubMed and Web of Science, with the last search date being July 8, 2026. The search covered studies on the epidemiology, diagnosis, molecular mechanisms, and AKR1B10-related research of malignant liver tumors in adults and children, and was limited to English-language publications.

The Key search terms included: “AKR1B10,” “aldose reductase-like protein 1,” “ARL-1,” “hepatocellular carcinoma,” “hepatoblastoma,” “pediatric liver cancer,” “alpha-fetoprotein,” “AFP,” “biomarker,” “diagnosis,” “prognosis,” “chemoresistance,” and others. The search strategy combined subject headings with free-text terms, using Boolean operators (AND, OR, NOT) to create combinations. For example, one of the search queries used in PubMed was: (AKR1B10 OR ARL-1) AND (hepatocellular carcinoma OR hepatoblastoma OR pediatric liver cancer) AND (diagnosis OR prognosis OR biomarker). Specific search queries were adjusted accordingly based on the syntactic characteristics of each database.

Literature selection followed the following principles: (1) priority was given to studies directly related to the expression, function, diagnostic value, and prognostic value of AKR1B10 in malignant liver tumors; (2) clinical studies, cellular and animal experiments on adult HCC, as well as relevant evidence on pediatric HB, pHCC, and rare pediatric liver tumors; (3) exclude duplicated publications, articles for which full-text access is unavailable, those with incomplete data, or literature unrelated to the topic of this review.

Regarding evidence grading, this review categorized the included literature into three types based on study design: adult clinical studies (including serological, histological, and prognostic studies), cellular and animal experiments (used to elucidate molecular mechanisms), and pediatric-related evidence (including clinical and basic research on HB, pHCC, and rare tumors). Given the extreme scarcity of direct studies on AKR1B10 in children, this review relies primarily on evidence from adults and theoretical extrapolation based on molecular overlaps between adult and pediatric liver tumors in the Wnt/β-catenin, lipid metabolism, and retinoic acid signaling pathways when inferring its potential value. It is explicitly noted that all pediatric-related applications are hypothetical and require further validation.

This review did not conduct new quantitative meta-analyses of the included literature nor employ systematic review or meta-analysis methods. All conclusions are based on a narrative synthesis and critical evaluation of the existing literature, aiming to provide a hypothetical framework and validation pathways for future research.

## 2 Epidemiological comparison and current diagnostic status

### 2.1 Incidence, disease burden, and pathological spectrum

Among adults, liver cancer remains the third leading cause of cancer-related deaths worldwide. According to GLOBOCAN 2022 data, there were nearly 20 million new cancer cases globally that year, with 9.7 million cancer-related deaths; liver cancer accounted for 7.8% of these deaths, second only to lung cancer (18.7%) and colorectal cancer (9.3%) (1). The risk factors for HCC vary by region. Chronic HBV/HCV infection accounts for 21%–55% of cases globally, while aflatoxin exposure, heavy alcohol consumption, overweight, type 2 diabetes, and smoking also contribute (1). Reports indicate that the number of liver cancer diagnoses is projected to continue rising through 2040 (22).

In contrast, primary malignant liver tumors in children are considered rare diseases. Data from the United States show that the annual incidence is approximately 2 cases per million children, accounting for only 0.5%–2% of all childhood malignancies (5). There are marked differences in the disease spectrum between children and adults: HB is the most common primary malignant liver tumor in children, accounting for approximately 80% of pediatric malignant liver tumors. The median age at diagnosis is 3 years, with the vast majority of cases occurring in children under 3 years of age (4, 5); pHCC is relatively rare, occurring primarily during adolescence, with an annual incidence of approximately 1 case per million children; 10%–50% of these cases have a recognizable genetic predisposition or metabolic disorder background (5, 8). According to a report by Hubbard et al., an upward trend in the incidence of hepatoblastoma has been observed in children under 5 years of age across multiple regions (23).

From the perspectives of histogenesis and molecular mechanisms, HB differs fundamentally from adult HCC. HB is an embryonic tumor originating from hepatoblasts, with a relatively low somatic mutation burden among human cancers (an average of approximately 3.9 mutations per tumor); the activating mutation in CTNNB1 is its core driving event (6, 7, 24–26). In contrast, adult HCC is characterized by a higher mutation burden and polygenic synergistic mutations—CTNNB1 mutations are observed in approximately 27% of HCC cases, and alterations in multiple other genes, including TP53, the TERT promoter, and AXIN1, are also involved (8, 16).

### 2.2 Limitations of existing serum biomarkers

Serum AFP combined with abdominal ultrasound is currently the most widely used noninvasive screening protocol for liver cancer (10). However, numerous studies have demonstrated that existing serological markers have significant limitations in the screening for early-stage liver cancer.

In adults with HCC, AFP has low sensitivity for detecting early-stage HCC (27), at only 44.4%, with a specificity of 84.8% and an area under the curve (AUC) of 0.52 (28). The sensitivity of abdominal ultrasound for early-stage HCC is approximately 45% (12). Even when ultrasound is combined with AFP, the sensitivity for detecting HCC can only be increased to approximately 63%, and the specificity is lower than that of ultrasound alone (12). More challenging still, 30%–40% of HCC cases and 60%–70% of early-stage HCC cases are AFP-negative (28, 29). Furthermore, it has been reported that AFP may yield false-positive results in certain conditions, such as cirrhosis (30).

Apart from AFP, other serum markers also have certain limitations. Serum alpha-fetoprotein heterogenin (lens culinaris agglutinin-reactive fraction of AFP, AFP-L3) is a commonly used AFP heterogenin subtype in clinical practice, but multiple studies have suggested that its contribution to HCC screening is limited. In a prospective evaluation of patients with chronic hepatitis B, the diagnostic performance of GAAD (Cobas) was non-inferior to that of GALAD (Cobas) and GALAD (µTASWAKO) (*p* < 0.001), suggesting that AFP-L3 plays a negligible role in the screening for hepatocellular carcinoma (31). Another study also confirmed that adding AFP-L3 to the combination of AFP and PIVKA-II does not further improve the ability to distinguish between HCC and cirrhosis (32). Although abnormal prothrombin [protein induced by vitamin K absence/antagonist-II (PIVKA-II) or des-gamma-carboxyprothrombin (DCP)] has shown diagnostic potential superior to that of AFP in some studies, its sensitivity for detecting early-stage micropatological HCC remains insufficient. Early studies showed that the DCP positivity rate in microhepatocellular carcinomas with a diameter ≤2 cm was only about 3% (33), and the sensitivity of DCP was also lower than that of AFP in small HCCs with a diameter <3 cm (34). In addition, factors such as liver function status, vitamin K deficiency, and warfarin use can all lead to false-positive DCP results (35). Overall, although derived markers such as AFP-L3 and DCP compensate to some extent for the limitations of AFP, their diagnostic performance for early-stage and AFP-negative HCC remains limited, and each has fundamental issues such as insufficient specificity or susceptibility to interference from clinical factors (36, 37).

### 2.3 Imaging studies

Imaging studies are a cornerstone of HCC diagnosis, but they have their own limitations in both adults and children. In adults, abdominal ultrasound, as a first-line screening tool, has a sensitivity of only approximately 45% for early-stage HCC, and the results are highly dependent on the operator's experience (38). Although CT and MRI offer higher spatial resolution, their high cost and limited accessibility make them unsuitable for large-scale screening.

The challenges of imaging diagnosis are particularly unique to children. Malignant liver tumors in children are relatively rare, and radiologists have limited opportunities to gain experience; therefore, diagnosing malignant liver tumors in children is highly challenging (39).

### 2.4 Specific diagnostic challenges in pediatric hepatoblastoma

The diagnosis of HB presents another specific challenge: physiologically elevated AFP levels in infants and young children. AFP is a glycoprotein produced by the fetal liver and embryonic yolk sac. After birth, its levels decline rapidly, typically reaching adult levels within 6–12 months. However, in children under 3 years of age—particularly infants under 1 year old—the normal range for serum AFP varies greatly from child to child and changes with age (40, 41). A study by Saraf et al. on the prediction of liver disease using a machine learning framework confirmed that the predictive ability of liver-related markers—such as alkaline phosphatase, bilirubin, transaminases, and albumin—varies significantly by age and sex, suggesting that age stratification must be considered when determining diagnostic thresholds for biomarkers (42).

Physiological elevation poses two challenges in the diagnosis of HB. The first challenge is that AFP levels in children with HB are often significantly elevated, with peaks reaching up to 1,000,000 ng/mL. However, in subtypes such as small-cell undifferentiated hepatoblastoma, AFP levels may be normal or only mildly elevated, which can easily lead to missed diagnoses (6). The second challenge is the lack of standardized, age-stratified AFP reference ranges. Consequently, it is difficult to distinguish between AFP elevations caused by benign liver disease or physiological states and those caused by HB. This may lead to unnecessary needle biopsies, delayed treatment, or even misdiagnosis (43). Therefore, in pediatric hepatoblastoma, alpha-fetoprotein (AFP) cannot be used as an independent diagnostic marker. It can be used for diagnostic evaluation, risk stratification, treatment monitoring, and assessment of recurrence; however, it must be interpreted in conjunction with age, imaging studies, PRETEXT staging, pathological findings, and other clinical observations. Two notable challenges are the physiologically elevated levels of alpha-fetoprotein in infants and the low levels of alpha-fetoprotein observed in small-cell undifferentiated hepatoblastoma.

AFP expression in HB is tightly regulated by the Wnt-β-catenin signaling pathway. Approximately 80% of HB cases involve mutations in the β-catenin gene, leading to persistent activation of this pathway and, consequently, driving AFP production. AFP expression patterns vary significantly across different histological subtypes. The hepatocellular subtype generally exhibits high AFP levels, often indicating better differentiation and a relatively favorable prognosis. In contrast, the mesenchymal subtype and the small-cell undifferentiated variant typically show very low or undetectable AFP levels, reflecting more aggressive molecular characteristics (15). These differences make AFP less reliable when used as a standalone diagnostic marker. To clearly illustrate the differences between adults and children regarding diagnostic challenges in AFP, this review summarizes the key points above as follows (Table 1).

**Table 1:** Comparison of diagnostic challenges with AFP in malignant liver tumors in adults and children

Dimensions	Adult HCC	Pediatric HB
Early detection rate	Early HCC detection rate is only 44.4%, with a high risk of missed diagnosis	Physiological elevations in AFP occur during infancy and early childhood, making it difficult to define a normal threshold
Proportion of AFP-negative cases	30%–40% of HCC cases are AFP-negative and require reliance on other biomarkers	Small-cell undifferentiated HB exhibits low AFP expression (<100 ng/mL), making it prone to being missed
Specificity and interferences	Liver diseases such as cirrhosis can cause false-positive AFP results, leading to insufficient specificity	AFP expression varies significantly across different histological subtypes, reducing the reliability of AFP when used alone
Common challenges	Early diagnosis is challenging across the board, creating an urgent need to identify new, complementary biomarkers	

AFP has significant diagnostic limitations in both adult and pediatric liver malignancies. In adult HCC, the main issues are low early detection rates, a high proportion of AFP-negative cases, and false-positive associated with cirrhosis. In pediatric HB, challenges include physiologically elevated AFP levels, missed diagnoses of low-AFP subtypes, and histological heterogeneity. HCC, hepatocellular carcinoma; HB, hepatoblastoma; AFP, alpha-fetoprotein.

### 2.5 Summary

In summary, although malignant liver tumors in adults and children differ significantly in etiology, pathogenesis, and pathological spectrum, the limiting role of early diagnosis in improving prognosis is common to both. The current serum biomarker AFP lacks sufficient sensitivity in adult HCC (particularly for early-stage and AFP-negative HCC), and other serum biomarkers also have certain limitations. In pediatric HB, diagnosis is further complicated by physiologically elevated AFP levels, and the heterogeneous AFP expression across different histological subtypes further limits its diagnostic value. Imaging tests also face issues such as limited sensitivity, high cost, or insufficient specificity. This diagnostic dilemma makes the exploration of novel, sensitive, and specific serum biomarkers an urgent priority. AKR1B10, a protein that is frequently overexpressed in HCC tissues and secreted into the bloodstream, has demonstrated diagnostic potential superior to that of AFP in adult studies; its value in pediatric liver malignancies warrants further systematic investigation.

## 3 Molecular basis and oncogenic mechanisms of AKR1B10

### 3.1 Gene and protein structure

AKR1B10 (Aldo-keto reductase 1B10), also known as Aldose Reductase-like Protein 1 (ARL-1), belongs to the Aldo-keto reductase (AKR) superfamily along with AKR1B1 and AKR1B15. This superfamily is divided into six distinct subfamilies: AKR1A, AKR1B, AKR1C, AKR1E, AKR6A, and AKR7A. They share a common set of structural features: an (*α*/β)_8_ barrel structure (also known as a TIM barrel), a disordered loop region, a cofactor-binding site, and a catalytic tetrapeptide (44–46). Members of the AKR superfamily are predominantly cytoplasmic monomeric proteins that use reduced nicotinamide adenine dinucleotide phosphate (NAD(P)H) as a cofactor to catalyze carbonyl reduction reactions (44, 47).

The human AKR1B subfamily consists of three members: AKR1B1, AKR1B10, and AKR1B15, all of which are located on chromosome 7q33 (45, 48). AKR1B10 shares 70% amino acid sequence homology with AKR1B1 (46, 49, 50) and as much as 91.5% homology with AKR1B15 (48). This protein consists of 316 amino acids, has a molecular weight of approximately 36 kDa, and exists as a soluble monomer in the cytoplasm (48). Its active site is located above the (*α*/β)_8_ barrel structure and contains a conserved catalytic tetrad (designated as Asp43, Tyr48, Lys77, and His110 in the AKR1B1 nomenclature), as well as a geometrically relatively rigid “anion-binding pocket” (ABP) (46, 48).

### 3.2 Physiological functions and tissue distribution

The expression of AKR1B10 in normal human tissues is selective. At the mRNA level, this gene is primarily expressed in the small intestine and colon, with lower levels in the liver (49). At the protein level, AKR1B10 is abundant in epithelial cells of the stomach, small intestine, and colon, whereas its expression is low or absent in other tissues, such as the liver, thymus, and prostate (48, 51).

Functionally, AKR1B10 is an NADPH-dependent reductase capable of catalyzing the reduction of various endogenous and exogenous aldehydes, ketones, and carboxylic acids. Its physiological substrates include: retinaldehyde (all-trans retinaldehyde, 9-cis-retinaldehyde,13-cis-retinal), cytotoxic aldehydes generated by lipid peroxidation (such as 4-hydroxy-nonenal, 4-HNE, and 4-oxo-2-nonenal, 4-ONE), as well as isopentenal, and others (47, 48, 50, 52). Among all members of the AKR1B subfamily, AKR1B10 exhibits the highest catalytic efficiency for retinal and is currently the most potent retinal reductase known (53). In the normal gastrointestinal tract, this enzyme exerts a detoxifying effect by reducing reactive aldehydes from food sources to their corresponding alcohols, thereby protecting mucosal epithelial cells from damage caused by carbonyl compounds (48, 49). This enzyme also exerts an anti-apoptotic effect by upregulating the expression of acetyl-CoA carboxylase *α* (ACCA), thereby promoting lipid synthesis and reducing mitochondrial membrane damage (54).

### 3.3 Expression patterns in hepatocellular carcinoma

The expression of AKR1B10 in HCC exhibits dynamic changes. The expression level of AKR1B10 is closely associated with the degree of differentiation and tumor stage of HCC. AKR1B10 is highly expressed in early-stage, well-differentiated HCC; however, its expression decreases in advanced, poorly differentiated stages (55–57). This phenomenon of “elevated expression in early stages and decreased expression in advanced stages” suggests that the upregulation of AKR1B10 is likely an early step in the development of HCC, playing a role in tumor initiation; once the tumor progresses to a high-grade stage and loses its differentiation characteristics, its expression subsequently decreases (51, 56, 58).

### 3.4 Core molecular mechanisms driving the development and progression of hepatocellular carcinoma

Studies have shown that AKR1B10 participates in the development and progression of HCC through multiple signaling and metabolic pathways. First, it catalyzes the reduction of retinaldehyde to retinol, thereby inhibiting retinoic acid (RA) production, which reduces RA-dependent differentiation signals and promotes cellular dedifferentiation. Second, AKR1B10 stabilizes the ACC*α* protein, enhances fatty acid synthesis, and supports tumor energy metabolism. Furthermore, it regulates signaling pathways, such as Wnt/β-catenin and PI3K/AKT, promoting HCC cell proliferation and migration.

#### 3.4.1 Regulation of retinoic acid metabolism and inhibition of cell differentiation

AKR1B10 catalyzes the reduction of retinaldehyde to retinol, a reaction that competes with the RA synthesis pathway. RA is a key signaling molecule that maintains cell differentiation by regulating the transcription of target genes through its binding to nuclear receptors. When AKR1B10 is overexpressed, the conversion of retinaldehyde to retinol is enhanced, reducing the availability of substrates for the oxidative generation of RA. Consequently, RA levels decrease, thereby inhibiting cell differentiation and promoting proliferation (48, 53, 55, 59, 60). Ruiz et al. demonstrated that the enzymatic activity of AKR1B10 reduces all-trans-RA- and 9-cis-RA-dependent trans-activation in living cells, suggesting that this enzyme is involved in the regulation of RA nuclear receptor precursors and that its overexpression may promote dedifferentiation and tumor development (61).

#### 3.4.2 Lipid metabolism reprogramming

In breast cancer cells, AKR1B10 promotes fatty acid synthesis by stabilizing ACCA(this mechanism has not yet been directly confirmed in hepatocellular carcinoma). ACCA is the rate-limiting enzyme in *de novo* fatty acid synthesis. In breast cancer cells, AKR1B10 binds to ACCA, preventing its ubiquitination and proteasomal degradation, thereby increasing ACCA protein levels and enhancing fatty acid synthesis (47, 51, 62). The liver is the central site for lipid processing and metabolism. Excessive lipid accumulation in the liver impairs liver function, gradually leading to fatty liver disease, followed by cirrhosis, and ultimately progressing to HCC (51). Furthermore, in liver cancer cells, AKR1B10 can also promote cell proliferation by regulating sphingosine-1-phosphate (S1P) signaling. Studies have found that levels of both AKR1B10 and S1P are higher in HCC tissues than in peritumoral tissues, and that the AKR1B10-S1P signaling axis in co-culture systems can enhance the proliferative capacity of hepatocytes (63).

#### 3.4.3 Regulation of signaling pathway networks

AKR1B10 is involved in the regulation of multiple oncogenic signaling pathways. Liu et al. reported that 14-3-3ε induces AKR1B10 expression by activating the β-catenin signaling pathway, whereas knocking out AKR1B10 inhibits 14-3-3ε-induced HCC cell proliferation and tumor growth *in vivo*. Silencing AKR1B10 also increases RA levels in mouse serum (17). Zhang et al. found that E2F1 transcriptionally upregulates AUF1, which stabilizes AKR1B10 transcripts by binding to the 3′UTR of AKR1B10 mRNA, thereby upregulating AKR1B10 expression and promoting HCC development and doxorubicin resistance (64). Cheng et al. identified AKR1B10 as a novel downstream target of IRAK1. IRAK1 upregulates AKR1B10 via the AP-1 signaling pathway, thereby regulating the self-renewal, tumor-forming capacity, and drug resistance of hepatocellular carcinoma stem cells. Inhibition of IRAK1/4 in combination with sorafenib synergistically suppresses the growth of HCC xenografts (65). Liu et al. also found that epidermal growth factor (EGF) and insulin can induce the transcriptional upregulation of AKR1B10 in hepatocellular carcinoma cells by activating the AP-1 signaling pathway, suggesting that this pathway may be involved in the abnormal expression of AKR1B10 during the development of hepatocellular carcinoma (66). Furthermore, AKR1B10 can promote the proliferation, migration, and invasion of hepatocellular carcinoma cells by activating the phosphorylation levels of PI3K and AKT (51).

#### 3.4.4 Chemotherapy resistance

Recent studies have shown that AKR1B10 can contribute to resistance to targeted therapies through metabolic reprogramming. In lenvatinib-resistant hepatocellular carcinoma, AKR1B10 drives resistance by enhancing aerobic glycolysis; this mechanism involves AKR1B10 lactylation mediated by alanine-tRNA synthetase 1 (AARS1), which stabilizes the AKR1B10 protein and promotes its interaction with lactate dehydrogenase A (LDHA), forming a positive feedback loop between AKR1B10, lactate, and LDHA that continuously drives glycolysis and drug resistance. Treatment with the AKR1B10 inhibitor, epalrestat, can effectively reverse lenvatinib resistance (67). In addition, in gastrointestinal cancer cells, AKR1B10 may also contribute to chemotherapy resistance through its carbonyl detoxification function. This enzyme reduces the carbonyl metabolites of chemotherapeutic agents, such as doxorubicin and mitomycin C, to their corresponding alcohols, thereby reducing the cytotoxic effects of the drugs (68). Regarding cisplatin, AKR1B10 does not act by directly reducing cisplatin itself, but rather by inhibiting the accumulation and cytotoxicity of 4-hydroxynonenal (4-HNE) and its cytotoxicity following cisplatin treatment, while simultaneously downregulating peroxisome proliferator-activated receptor *γ* (PPAR*γ*), thereby mediating drug resistance; however, the use of AKR1B10 inhibitors in combination with PPAR*γ* ligands can restore sensitivity to cisplatin (CDDP) (68). Other reports have also indicated that AKR1B10 is significantly upregulated in certain cancer cell lines that have acquired resistance to cyclophosphamide and doxorubicin (69). Taking these four pathways into account, AKR1B10 forms a complex regulatory network in the development and progression of hepatocellular carcinoma through the regulation of retinoic acid metabolism, lipid metabolism reprogramming, regulation of signaling networks, and chemoresistance (Figure 1).

**Figure 1:**
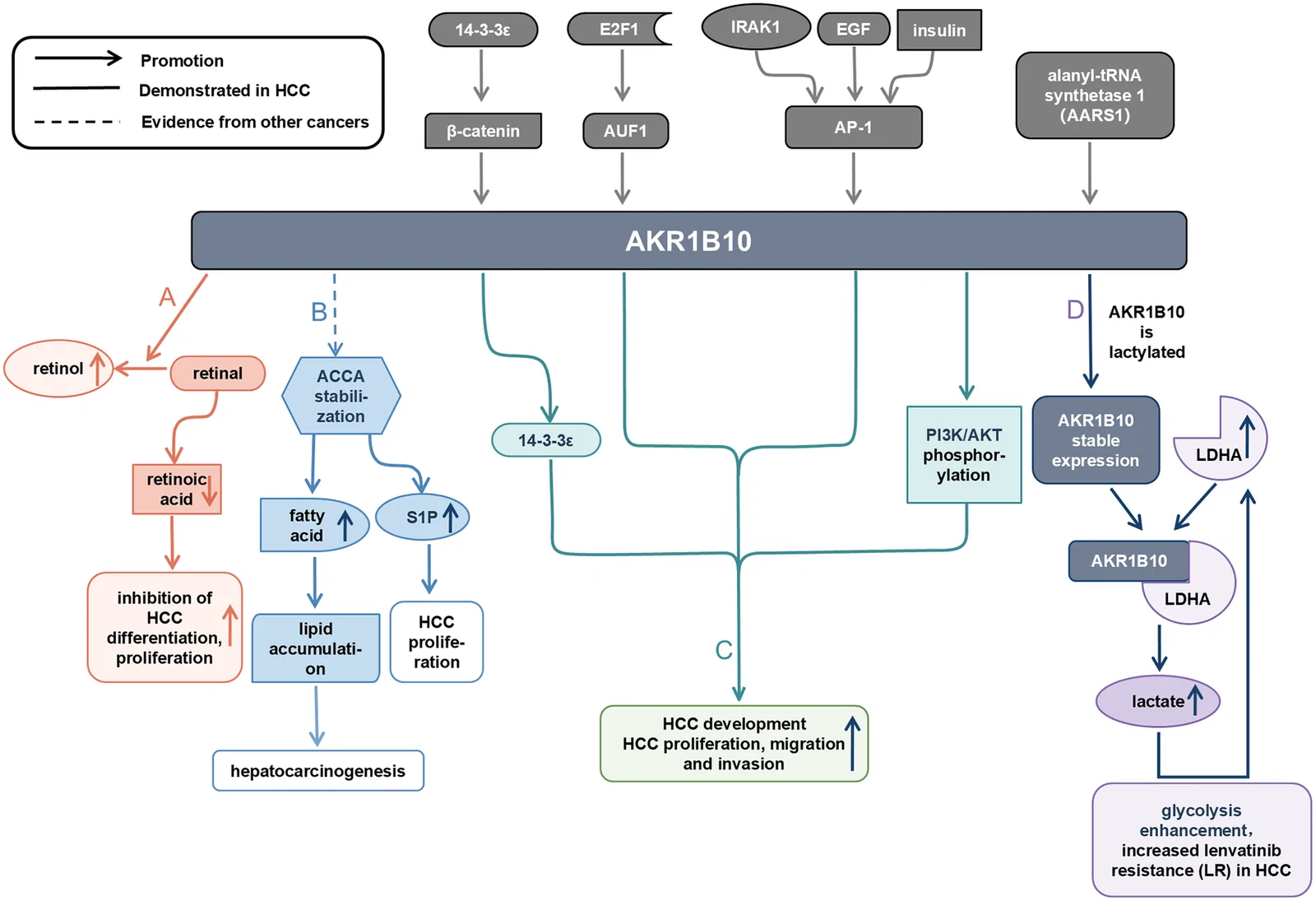
Network diagram of the core molecular mechanisms by which AKR1B10 drives the onset and progression of hepatocellular carcinoma. The figure illustrates how AKR1B10 participates in the onset and progression of hepatocellular carcinoma through four core pathways. **(A)** Regulation of retinoic acid metabolism. AKR1B10 catalyzes the reduction of retinaldehyde to retinol, leading to decreased RA levels, which inhibits cell differentiation and promotes proliferation. **(B)** Lipid metabolism reprogramming. AKR1B10 stabilizes ACCA, promoting fatty acid synthesis, and facilitates cell proliferation via S1P signaling. **(C)** Regulation of signaling pathway networks. AKR1B10 participates in multiple signaling pathways, including 14-3-3ε/β-catenin, E2F1/AUF1, IRAK1/AP-1, and EGF/AP-1, promoting the proliferation, migration, and invasion of hepatocellular carcinoma cells. **(D)** Chemoresistance. AKR1B10 enhances aerobic glycolysis through lactylation, forming an AKR1B10-lactate-LDHA positive feedback loop that drives lenvatinib resistance. ↑: Upregulated or promoted; ↓: Downregulated or inhibited. AKR1B10, Aldo-keto reductase 1B10; RA, retinoic acid; ACCA, acetyl-CoA carboxylase *α*; S1P, sphingosine-1-phosphate; LDHA, lactate dehydrogenase A.

It should be noted that AKR1B10 may also play a compensatory protective role in the development of HCC. Liu et al. found that during the progression of chronic liver disease, increased oxidative stress upregulates AKR1B10 via NRF2, and AKR1B10, through its redox and detoxification activities, protects hepatocytes from reactive oxygen species (ROS) damage. Conversely, AKR1B10 deficiency accelerates the development of HCC associated with hepatotoxins and inflammation, suggesting that elevated AKR1B10 expression in HCC may partly represent a compensatory response to oxidative stress (70).

### 3.5 Sensitivity basis as a “mechanism-driven” serum biomarker

AKR1B10 possesses several characteristics that give it the potential to serve as an ideal serum biomarker. **Feature 1:** It is a secreted protein. Studies have confirmed that AKR1B10 is secreted from normal intestinal epithelial cells and cancer cells via a lysosome-mediated non-classical pathway. Its concentration in ileal fluid can reach 188.6–535.7 ng/mL. This suggests that the protein can enter the bloodstream, making it a potential serum biomarker (71). **Feature 2:** AKR1B10 expression in HCC tissue exhibits a dynamic pattern characterized by upregulation in the early stages and downregulation in the late stages. Therefore, it has high sensitivity for detecting early-stage HCC (55–57). **Feature 3:** Large-scale, multicenter clinical studies have validated the diagnostic value of serum AKR1B10. A study by Ye et al. involving a multicenter cohort showed that the AUC for serum AKR1B10 in detecting HCC was superior to that of AFP. This advantage was even more pronounced in early-stage HCC and AFP-negative HCC. The combined use of AKR1B10 and AFP can further improve diagnostic accuracy (13, 14). A meta-analysis indicated that the overall specificity of AKR1B10 for diagnosing HCC was 78%, with a sensitivity of 85%. Its diagnostic performance for early-stage HCC surpasses that of AFP, and since serum levels decline rapidly after surgery, it can be used to monitor treatment response (72). A recent case report showed that serum AKR1B10 levels were significantly elevated in patients with hepatitis B-associated HCC who remained persistently AFP-negative. This suggests that it can serve as a complementary marker for AFP-negative HCC (9).

Overall, AKR1B10 has established a relatively comprehensive chain of evidence ranging from its molecular structure, physiological functions, and abnormal expression patterns to its carcinogenic mechanisms and clinical diagnostic efficacy. This lays a solid foundation for its use as a novel biomarker and potential therapeutic target in HCC.

## 4 Diagnostic and clinical value of AKR1B10 in adult hepatic malignancies

### 4.1 The value of serum AKR1B10 in early screening, postoperative monitoring, and early warning

As discussed in the previous section, several large-scale clinical studies have validated the diagnostic value of serum AKR1B10 as a marker for HCC, and AKR1B10 has also demonstrated a unique complementary value for clinically challenging AFP-negative HCC. Gao et al. reported two cases of hepatitis B-related HCC in which the patients’ serum AFP levels remained persistently negative (<20 ng/mL), but serum AKR1B10 levels were significantly elevated (reaching 6,239.29 pg/mL and 1,500.00 pg/mL, respectively), suggesting that AKR1B10 can serve as a powerful auxiliary diagnostic marker for AFP-negative HCC (9).

AKR1B10 also holds potential for postoperative monitoring of HCC. Studies have shown that serum AKR1B10 levels decline more rapidly than AFP following radical tumor resection, which aids in assessing the extent of surgical resection and the early detection of postoperative recurrence (13, 72).

Furthermore, AKR1B10 can be used to predict the risk of HCC development in high-risk populations. A study by Murata et al. in patients with chronic hepatitis C (CHC) found that patients with high AKR1B10 expression (≥8%) in the liver prior to treatment had a risk of subsequent HCC development approximately 15 times higher than those with low AKR1B10 expression, even after achieving sustained virologic response (SVR) through antiviral therapy. The negative predictive value of this study was as high as 0.991, suggesting that baseline AKR1B10 expression can be used to identify patients with extremely low HCC risk among those who achieved SVR, thereby avoiding unnecessary frequent monitoring (73) (Supplementary Table S1).

### 4.2 Tissue AKR1B10 for differential diagnosis and prognostic assessment

In addition to serum testing, AKR1B10 expression at the tissue level is valuable for the differential diagnosis and prognostic assessment of liver tumors. Immunohistochemical studies have found that AKR1B10 is overexpressed in 97% of HCC tissues, whereas it is virtually absent in benign liver lesions, such as hepatic adenomas and focal nodular hyperplasia, suggesting that tissue AKR1B10 staining aids in distinguishing HCC from benign liver lesions (55).

Regarding prognosis, some studies suggest that high AKR1B10 expression indicates favorable prognosis. Liu et al. reported that among 14-3-3ε-positive HCC patients, those with increased AKR1B10 expression had higher overall survival and disease-free survival rates, as well as a lower incidence of metastasis (17). Wang et al. also found that high AKR1B10 expression was associated with a reduced risk of early postoperative recurrence and improved overall survival in patients with HBV-associated HCC (74). Heringlake et al. observed that AKR1B10 was overexpressed in the early stages of well- and moderately differentiated tumors but downregulated in poorly differentiated, advanced tumors, suggesting that its expression may be related to the degree of differentiation (56). However, other studies have reached opposite conclusions. Ma et al. found that elevated AKR1B10 expression in HCC tissues was significantly associated with patient sex, primary tumor diameter, and the stage of liver fibrosis; high expression indicated a poor prognosis and could serve as an independent prognostic marker for assessing clinical outcomes in HCC patients (57). Xie et al. also reported that serum AKR1B10 levels were associated with poor survival in patients and were superior to AFP in prognostic assessment (75).

Possible reasons for these discrepancies include differences in patient inclusion criteria across studies (e.g., HBV-associated vs. non-viral HCC), sample size, and statistical methods, as well as the dual role AKR1B10 may play in the development of liver cancer—exercising a protective effect in the early stages through NRF2-mediated compensatory upregulation, while driving tumor progression in the late stages through mechanisms such as promoting lipid synthesis, retinoic acid depletion, and chemoresistance (70). Therefore, the prognostic significance of AKR1B10 may be influenced by multiple factors, including etiology, tumor stage, and clinical context; its prognostic value must be interpreted in the context of specific clinical situations.

### 4.3 Extended applications in intrahepatic cholangiocarcinoma, cHCC-CCA, and other rare tumors

In addition to HCC, AKR1B10 has also been studied in other malignant liver tumors. In cholangiocarcinoma (CCA), immunohistochemical analysis showed that AKR1B10 expression was upregulated in well-differentiated early-to-mid-stage tumors and downregulated in poorly differentiated late-stage tumors, suggesting that it may serve as a potential biomarker for CCA (56). Cai et al. revealed that METTL3 promotes glycolysis and tumor progression in CCA by mediating m6A modification of AKR1B10; knocking down AKR1B10 reverses the pro-tumor effects caused by METTL3 overexpression (76).

Currently, no studies have specifically addressed AKR1B10 expression or its diagnostic value in combined hepatocellular-cholangiocarcinoma (cHCC-CCA). However, this tumor exhibits pathological features of both HCC and CCA, and the Wnt/β-catenin pathway is activated in it (77, 78). Given that AKR1B10 is abnormally expressed in both HCC and CCA, its potential application in cHCC-CCA requires further investigation.

Overall, research on AKR1B10 in rare tumors, such as intrahepatic cholangiocarcinoma and cHCC-CCA, is still in its early stages, and systematic validation in clinical cohorts is lacking (51). To systematically outline the complete research trajectory of AKR1B10, from molecular cloning to clinical application, this review summarizes the key milestones in a timeline (Figure 2).

**Figure 2:**
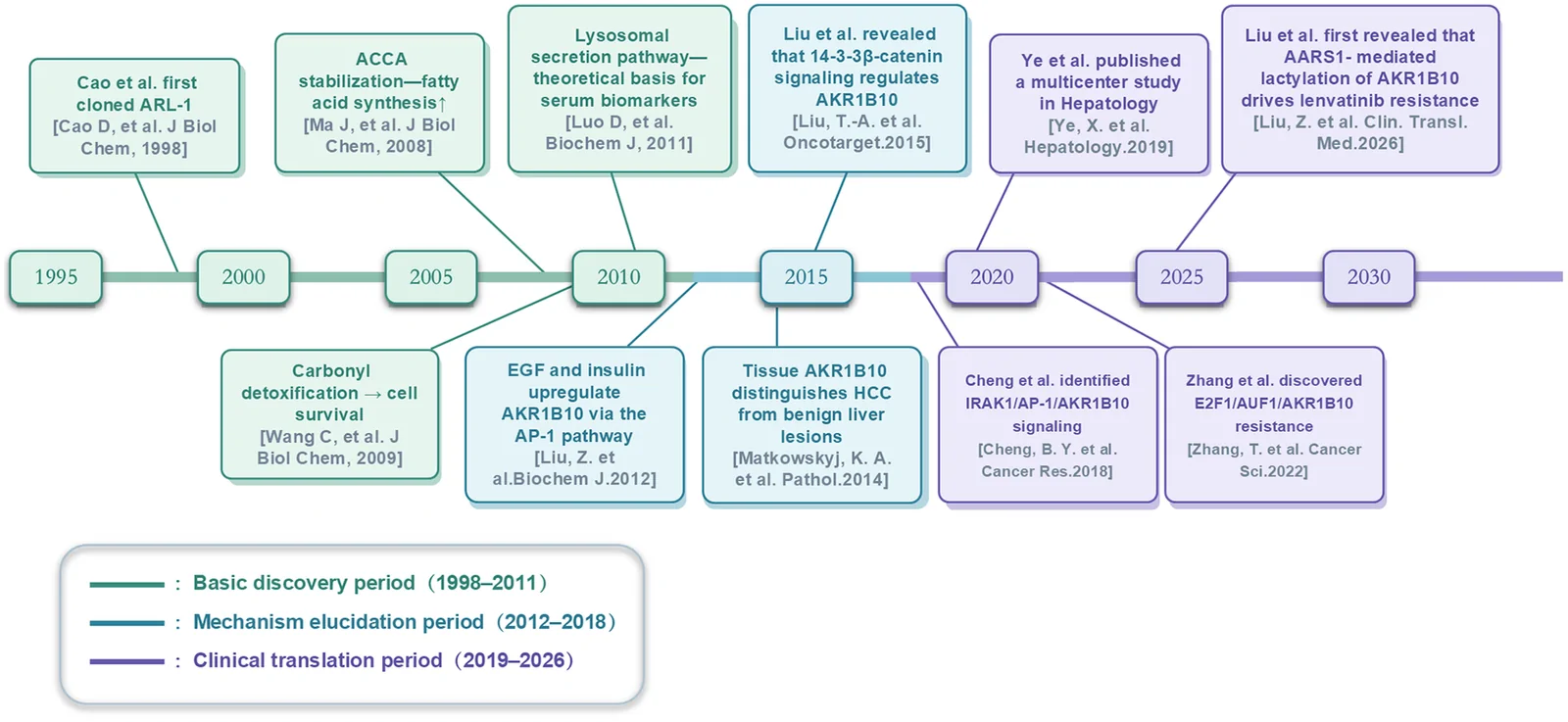
Timeline of research on AKR1B10 as a serum marker for HCC. This figure summarizes the major research milestones for AKR1B10 since its initial cloning and identification in 1998. The research journey can be divided into three phases: Basic Discovery Phase (1998–2011)—elucidation of molecular cloning, physiological functions, and secretion pathways; Mechanism Elucidation Phase (2012–2018)—revelation of multiple signaling pathways and carcinogenic/drug-resistance mechanisms; Clinical Translation Phase (2019–2026)—multicenter clinical validation and expansion into the field of pediatric oncology. ACCA, acetyl-CoA carboxylase alpha.

## 5 From adult evidence to pediatric liver tumors

### 5.1 Several research directions worth noting in HB

HB is characterized by the activation of the Wnt/β-catenin pathway, and CTNNB1 mutations are present in approximately 70%–90% of cases. In adult HCC, AKR1B10 is transcriptionally regulated by β-catenin signaling (6, 15–20, 24, 79, 80). Based on this, it is hypothesized that AKR1B10 may also be upregulated in HB, and its expression levels may be associated with CTNNB1 mutation status and histological subtype. The histological subtypes of hepatoblastoma can be classified as epithelial or mixed epithelial-mesenchymal. The subtypes of epithelial hepatoblastoma include pure fetal type, embryonal type, small-cell type, and cholangioblastic type; different subtypes have distinct molecular characteristics and clinical prognoses (6). For example, pure fetal HB is often associated with large-segment deletions in exons 3–4 of CTNNB1, whereas the embryonic type is primarily characterized by small-segment mutations in exon 3 (81, 82). Therefore, the positive expression rate of AKR1B10 in HB tissues, its subcellular localization, and its relationship with different histological subtypes and differentiation levels remain to be further investigated.

Alpha-fetoprotein (AFP) remains an important biomarker for hepatoblastoma (HB), but its clinical significance must be evaluated in the context of specific clinical scenarios, as its role varies in screening, diagnostic evaluation, risk stratification, treatment monitoring, and assessment of recurrence. Interpretation of this marker must be based on a comprehensive evaluation that considers age, preterm birth, imaging findings, PRETEXT staging, pathological results, and the stage of treatment. On the one hand, serum AFP levels in healthy infants and young children rise physiologically after birth and do not approach adult levels until approximately 8 months of age, which may complicate the interpretation of AFP levels during infancy (40, 41, 83). On the other hand, serum AFP levels may be below 100 ng/mL in patients with small-cell undifferentiated HB and certain subtypes with low AFP expression; while such patients were previously considered to have a poor prognosis, recent studies suggest that, after ruling out malignant rhabdoid tumor (MRT), low AFP alone is not an independent factor for poor prognosis (6, 84). In adult HCC, serum AKR1B10 has a complementary diagnostic value for AFP-negative HCC, and its diagnostic performance is superior to that of AFP (9, 13). Therefore, it is necessary to verify whether, in pediatric HB patients, serum AKR1B10 can distinguish between pathological elevation and physiological fluctuations; whether AKR1B10 has adjunctive diagnostic potential for HB subtypes with elevated or low AFP expression (such as small-cell undifferentiated HB); and whether serum AKR1B10 also has supplementary diagnostic value for AFP-negative HB.

High-risk HB patients often require intensive chemotherapy regimens containing drugs such as cisplatin and doxorubicin; however, some patients exhibit primary or secondary drug resistance, leading to a poor prognosis (15, 85, 86). In adult HCC and other gastrointestinal tumors, AKR1B10 contributes to resistance to cisplatin and doxorubicin through carbonyl detoxification (64, 65, 68). Based on this, further investigation could explore whether AKR1B10 is upregulated in drug-resistant HB cells and whether its expression levels correlate with chemotherapy response in patients with HB (e.g., changes in PRETEXT staging, surgical resectability) and event-free survival.

### 5.2 Research gaps in pHCC

pHCC is rare. There are approximately 0.4–1.4 new cases per million children annually. In the 15–19 age group, it accounts for up to 87% of primary malignant liver tumors (5, 8). Approximately 10% to 50% of children with pHCC have a specific predisposing condition. Examples include hereditary tyrosinemia, perinatal HBV infection, familial intrahepatic cholestasis, glycogen storage disease, and congenital porto-systemic shunts. Furthermore, 26%–62% of these children do not have liver cirrhosis (5, 8). From a molecular perspective, pHCC shares similarities with adult HCC, such as abnormalities in pathways including Wnt/β-catenin, TP53, and the TERT promoter. However, the overall mutation burden in pediatric tumors is lower, and the presence of cirrhosis is less common (87).

Based on histological and molecular characteristics, pHCC can be classified into three categories: conventional HCC (histologically similar to that in adults), fibrolamellar carcinoma (FLC), and hepatocellular tumors with HB components, also known as “hepatocellular neoplasm not otherwise specified” (HCN NOS) (87). FLC accounts for 15%–25% of pediatric HCC cases. It predominantly affects adolescents, is not associated with cirrhosis, and typically presents with normal serum AFP levels; it is characterized by a distinctive DNAJB1::PRKACA fusion gene. This type of cancer is resistant to chemotherapy and has a relatively favorable prognosis, but complete surgical resection is essential (4, 5, 8). HCN NOS exhibits characteristics of both HB and HCC; it is more common in children of intermediate age, with a molecular profile intermediate between the two, and a relatively poor prognosis (4, 87). Currently, the treatment of pHCC primarily draws on experience with hepatoblastoma. The surgical resection rate is only approximately 27%. However, pediatric HCC responds better to chemotherapy than adult HCC, with an efficacy rate of approximately 39%. The outcomes of liver transplantation may actually be better in children who do not meet the Milan criteria (8).

Studies on adult HCC have demonstrated that AKR1B10 is a potential serum diagnostic marker, particularly in early-stage and AFP-negative cases (13, 72). However, the expression of AKR1B10 in pHCC has not yet been reported. Given the similarities in molecular pathways between pHCC and adult HCC, coupled with the fact that AFP levels are not elevated in some pHCC subtypes (such as FLC and HCN NOS), we hypothesize that AKR1B10 may also have diagnostic or adjunctive diagnostic value in pHCC.

### 5.3 Research needs for rare pediatric liver tumors

In addition to HB and pHCC, pediatric liver malignancies include undifferentiated embryonal sarcoma (UESL), epithelioid hemangioendothelioma (EHE), MRT, and angiosarcoma, which account for approximately 10%–15% of pediatric liver malignancies (39, 88, 89). Currently, there are no specific serum biomarkers for these tumors. For example, serum AFP levels in UESL are typically normal, and diagnosis relies primarily on imaging and histopathology (90, 91). In studies on adults, AKR1B10 is expressed in intrahepatic cholangiocarcinoma (ICC), and its expression level correlates with the degree of differentiation (56, 76). However, the expression status of AKR1B10 in rare pediatric liver malignancies remains entirely unknown. Therefore, it is necessary to investigate whether AKR1B10 is positively expressed in the aforementioned rare tumors and whether its expression has diagnostic or differential diagnostic value (e.g., distinguishing UESL from mesenchymal hamartomas). Given the extreme rarity of these cases, it is recommended that a sufficient number of tissue samples be collected through multicenter retrospective studies or pediatric cancer cooperative group biobanks to conduct preliminary immunohistochemical screening.

## 6 Discussion

In adult HCC, AKR1B10 contributes to tumor progression through three core pathways: Wnt/β-catenin signaling, lipid metabolism reprogramming, and retinoic acid metabolism. The presence of similar abnormalities in these pathways in pediatric liver malignancies (particularly HB and pHCC) is a prerequisite for hypothesizing the potential role of AKR1B10.

The Wnt/β-catenin signaling pathway plays a key role in HB. Approximately 70%–90% of HB cases harbor activating mutations in CTNNB1, leading to nuclear accumulation of β-catenin and sustained activation of downstream target genes (6, 7, 15, 24, 79, 80). In contrast, the prevalence of CTNNB1 mutations in adult HCC is approximately 35%, and the mutation profile differs (16, 18, 19). Although abnormalities in the Wnt pathway are present in both tumor types, the mutation frequency of this pathway is higher in HB and may occur at an earlier stage of tumor initiation (7).

There is substantial evidence of lipid metabolic reprogramming in adult HCC; AKR1B10 promotes fatty acid synthesis by stabilizing ACCA (47, 51, 62). Regarding HB, embryonic-type HB cells exhibit strong glycolytic characteristics and are sensitive to hexokinase-1 inhibition, suggesting that metabolic reprogramming is also involved in HB progression (81). However, there are currently no direct reports on the specific regulatory networks of lipid metabolism in HB, particularly regarding the involvement of AKR1B10.

Regulation of retinoic acid metabolism is another key mechanism by which AKR1B10 acts in adult HCC. This enzyme efficiently reduces retinaldehyde to retinol, leading to retinoic acid depletion, which inhibits differentiation and promotes proliferation (48, 53, 55, 59, 60). During liver development, retinoic acid signaling plays a crucial regulatory role in the proliferation and differentiation of hepatic stem/progenitor cells (92–96). As an embryonic tumor, HB development may be influenced by retinoic acid (RA) signaling. Previous studies have shown that the retinol X receptor (RXR) pathway is downregulated in metastatic HB cells, and its activation can inhibit HB cell invasion and stem cell properties (97). Furthermore, dysregulation of retinoic acid signaling is associated with various liver diseases, including HCC (98).

In summary, adult HCC and pediatric HB exhibit a certain degree of molecular overlap in the Wnt/β-catenin, lipid metabolism, and retinoic acid metabolism pathways; however, differences exist in the specific regulatory networks and mutation profiles. Based on this, the following key question is raised: whether AKR1B10 is expressed in pediatric liver malignancies (particularly HB and pHCC), and whether its upstream regulatory factors and downstream effector networks are consistent with those in adult HCC, remains to be further investigated.

To date, there have been no direct studies on the expression of AKR1B10 in pediatric liver tumors. AKR1B10 expression levels in adult HCC vary with the degree of differentiation: it is significantly upregulated in early, well-differentiated HCC and downregulated in late, poorly differentiated HCC (55–57). The frequency of Wnt/β-catenin pathway activation in pediatric HB (70%–90%) is higher than that in adult HCC (approximately 35%); however, hepatoblastoma (HB) is an embryonic tumor with a unique developmental origin and a low mutation burden, and its biological characteristics differ significantly from those of adult HCC. The presence of shared pathway abnormalities does not imply that the expression, regulation, secretion levels, or diagnostic utility of AKR1B10 are comparable (6, 7, 15, 16, 18, 19, 24, 79, 80). Therefore, all pediatric-related applications proposed in this paper remain purely hypothetical, the expression positivity rates, intensity, subcellular localization, and differences from adult HCC of AKR1B10 in HB, pHCC, and rare pediatric liver malignancies (such as UESL, EHE, MRT, and angiosarcoma) all require systematic characterization (39, 89). Furthermore, the major driving pathways in HB include Wnt/β-catenin, IGF2, and MYC (6, 7, 79). In adult HCC, AKR1B10 is regulated by β-catenin (17, 20); however, whether it is similarly regulated in HB and whether there are regulatory relationships with IGF2 and MYC remain to be verified. Normal ranges and physiological variations of serum AKR1B10 in children (especially infants and toddlers) have not yet been established. Serum AKR1B10 levels may be influenced by age, sex, puberty, inflammation, liver damage, renal function, obesity, and medications. Therefore, pediatric studies must utilize healthy control populations as well as children with benign or non-malignant liver disease to establish age- and sex-specific reference ranges. Multivariate models should account for the aforementioned confounding factors; adult cutoff values (e.g., ≥1,078.2 pg/mL for the diagnosis of nonalcoholic steatohepatitis) (99) cannot be directly extrapolated to pediatric populations and require independent validation. Xie et al. conducted transcriptomic and chromatin accessibility analyses of peripheral blood mononuclear cells from neonates and adults and found that neonates exhibited 669 overexpressed and 440 underexpressed genes, with as many as 36,782 peaks of differential chromatin accessibility, suggesting global differences in gene expression regulatory networks between adults and neonates (100). This further suggests that the mechanisms governing AKR1B10 expression in adult HCC and its diagnostic utility cannot be simply extrapolated to pediatric liver tumors but must be validated through direct testing using pediatric tissue samples.

Methodologically, IHC and RT-qPCR assays can be performed using existing pediatric liver tumor tissue banks or retrospective paraffin-embedded blocks, with stratified analysis by histological subtype (epithelial and mixed epithelial-mesenchymal; epithelial subtype: pure fetal type, embryonal type, small-cell type, cholangioblastic type, and epithelial-mesenchymal) (6, 81, 82). Case-control studies can be conducted using biobanks from pediatric tumor consortia (e.g., CHIC, PHITT) to preliminarily assess the diagnostic value of serum AKR1B10, considering the interference of physiologically elevated AFP levels in infants and young children on baseline assessments (84). HB cell lines (HepG2, Huh-6, and HepT1) and PDTX models (11, 101) can be used to study AKR1B10 function through knockdown/overexpression; combine AKR1B10 with AFP, PRETEXT staging, and histological subtypes to explore the feasibility of a multiparameter model (6, 13, 15, 72).

Regarding targeted therapy, in adults, the AKR1B10 inhibitor epalrestat enhances the anti-HCC effect of sorafenib (by blocking the mTOR pathway via) (102) and overcomes platinum resistance in non-small cell lung cancer organoids (103). In gastrointestinal tumors, inhibiting AKR1B10 can reverse cisplatin/doxorubicin resistance (68). When extending to pediatric HB, it is necessary to determine whether inhibiting AKR1B10 can enhance sensitivity to cisplatin/doxorubicin. Furthermore, the pharmacokinetics and safety of epalrestat in children remain unclear and must first be evaluated in PDTX models. Additionally, attention should be paid to the issue of selective off-target inhibition between AKR1B10 and the highly homologous AKR1B1 (70.89% sequence homology) (104, 105).

It should be clarified that the primary anticipated clinical application proposed in this review is to evaluate whether serum AKR1B10 can provide additional information in the diagnostic assessment of children suspected of having HB, building upon age-adjusted AFP levels and existing clinical evaluations. It is intended as an adjunctive marker, not an independent diagnostic tool, and is not suitable for large-scale screening in the general pediatric population. Treatment monitoring, prognostic assessment, and exploration of targeted therapies are secondary research directions that should be pursued only after the diagnostic value has been fully validated.

The serological study proposed in this research is currently at the hypothesis stage, and its validation pathway requires further refinement. Future studies should meet the following requirements: First, conduct formal analytical validation of the serum AKR1B10 assay to determine the limit of detection, precision, linear range, and inter-batch variability; Second, establish an age-matched pediatric control group, including healthy children and those with benign or non-malignant liver disease, to establish age- and sex-specific reference ranges; Third, validate the diagnostic performance in an independent pediatric cohort to avoid single-center bias; Fourth, assess the incremental diagnostic value of AKR1B10 compared to age-adjusted AFP and existing clinical variables (such as PRETEXT staging, imaging features, and histological subtypes). The above requirements were incorporated into the phased validation framework presented in Table 2. Furthermore, diagnostic thresholds established for adults cannot be directly extrapolated to children; they must be independently established based on pediatric data.

**Table 2:** Proposed phased, exploratory validation framework for AKR1B10 in pediatric liver tumors.

Stage	Objective	Design/population	Key methods/samples	Main endpoints/analyses
1	Characterize AKR1B10 expression in HB and pediatric controls	Retrospective tissue study; HB and pediatric controls (benign, non-malignant, other tumors)	IHC, RT-qPCR; stratified by histological subtype and, if available, CTNNB1 status	Positivity rate, expression intensity, localization; preliminary association with PRETEXT, metastasis, AFP
2	Establish age- and sex-specific serum reference ranges	Cross-sectional; healthy children and children with benign/non-malignant liver disease	Serum AKR1B10 ELISA; age/sex stratification; formal analytical validation (limit of detection, precision, linearity, inter-assay variation)	Reference intervals; physiological variation; comparison with adult cutoffs
3	Assess diagnostic performance as an adjunct to age-adjusted AFP	Case-control; children undergoing diagnostic evaluation for suspected HB; pediatric tumor consortium biobanks (e.g., CHIC, PHITT)	Serum AKR1B10 and AFP ELISA; compare AKR1B10 alone, AFP alone, and combination; independent validation in a separate pediatric cohort	ROC/AUC, sensitivity, specificity; incremental diagnostic value beyond age-adjusted AFP and established clinical variables (e.g., PRETEXT, imaging, histology)
4	Explore associations with treatment response, recurrence, and chemoresistance (secondary, exploratory)	Longitudinal observational; pediatric HB patients; exploratory HB cell lines and PDTX models	Serial serum ELISA; knockdown/overexpression and drug-sensitivity assays	Dynamic changes; association with treatment response, recurrence, resistance

All proposed studies are exploratory and hypothesis-generating and do not constitute clinically validated applications. AKR1B10 is proposed as an adjunctive, rather than a standalone, biomarker. Adult diagnostic thresholds should not be directly extrapolated to pediatric populations. Stage 1: Tissue expression characterization in HB and relevant pediatric controls. Stage 2: Age- and sex-specific serum reference ranges in children. Stage 3: Case-control assessment of diagnostic performance, comparing AKR1B10 alone, AFP alone, and their combination. Stage 4: Exploratory evaluation of associations with treatment response, recurrence, and chemoresistance, including mechanistic studies in HB cell lines and PDTX models. All proposed studies are exploratory and hypothesis-generating and do not constitute clinically validated applications. AKR1B10, Aldo-keto reductase 1B10; HB, hepatoblastoma; AFP, alpha-fetoprotein; IHC, immunohistochemistry; RT-qPCR, reverse transcription quantitative PCR; ELISA, enzyme-linked immunosorbent assay; ROC, receiver operating characteristic; AUC, area under the curve; PDTX, patient-derived tumor xenograft; CHIC, Children's Hepatic Tumors International Collaboration; PHITT, Pediatric Hepatic International Tumor Trial.

Based on the above analysis, we propose a phased exploratory framework for future pediatric studies (Table 2). This framework comprises four stages: (1) characterization of AKR1B10 expression in HB tissues and relevant pediatric controls, (2) establishment of age- and sex-specific serum reference ranges, (3) case-control assessment of diagnostic performance, and (4) exploratory evaluation of associations with treatment response, recurrence, and chemoresistance. These studies are intended to generate hypotheses and foundational data and do not constitute clinically validated applications.

## 7 Conclusion

This paper reviews the molecular mechanisms of AKR1B10 in adult hepatocellular carcinoma and its clinical diagnostic value, and attempts to extend the research approach for this biomarker to pediatric liver malignancies. To date, no studies have directly addressed AKR1B10 in pediatric liver tumors. The expression patterns of this molecule in pediatric liver tumors, its diagnostic potential, its relationship with chemotherapy resistance, and its specific role in rare tumors remain unknown. Based on the evidence accumulated in adult hepatocellular carcinoma, this paper proposes several testable hypotheses and suggests starting with tissue expression screening and preliminary serum analysis to gradually accumulate data. This paper aims to draw more researchers’ attention to this field and, through rigorous experimental design and the accumulation of evidence, gradually clarify the exact role that AKR1B10 plays in pediatric liver malignancies.
