# Supplementary material for: Diagnostic potential of Aldo-keto reductase 1B10 in pediatric hepatic malignancies: a narrative, hypothesis-generating translational review based on adult evidence and future research directions
Source: Front Med (Lausanne). 2026 Oct 23;13:1972682. doi: 10.3389/fmed.2026.1972682 (PMC13740262; doi:10.3389/fmed.2026.1972682)
Supplement: Supplementary file 1 [file Table1.docx]

**Supporting Table S1:** *Summary of major adult clinical evidence for AKR1B10 in HCC: diagnostic performance.* NR = not reported in the provided articles. TRFIA, time-resolved fluoroimmunoassay; HC, healthy control; BLT, benign liver tumor; CHB, chronic hepatitis B; LC, liver cirrhosis; BLD, benign liver disease; HA, hepatic adenoma; FNH, focal nodular hyperplasia; IHC, immunohistochemistry; ELISA, enzyme-linked immunosorbent assay; AUC, area under the curve.

| **Study (Ref)** | **Population** | **Control** | **Assay / sample** | **Cutoff** | **Sensitivity** | **Specificity** | **AUC** | **Early-stage HCC** | **AFP-negative HCC** | **Independent validation** | **Key outcome** | **Evidence level for pediatric application** |
| --- | --- | --- | --- | --- | --- | --- | --- | --- | --- | --- | --- | --- |
| Ye et al., 2019 (ref 13) | Large-scale multicenter; training cohort: 209 HCC, 203 HC, 57 BLT, 10 CHB, 40 LC; validation cohort: 204 HCC, 208 HC, 50 BLT, 22 CHB, 38 LC; follow-up cohort: 68 HCC | HC, BLT, CHB, LC | Serum AKR1B10 by TRFIA; AFP by ELISA | Training: AKR1B10 267.9 pg/mL; AFP 20.2 ng/mL | Training HCC vs all controls: AKR1B10 72.7%; AFP 65.1%; combination 84.2%. Validation: AKR1B10 72.1%; AFP 67.6%; combination 77.0% | Training: AKR1B10 95.7%; AFP 88.9%; combination 90.1%. Validation: AKR1B10 90.7%; AFP 80.7%; combination 93.5% | Training HCC vs all controls: AKR1B10 0.896; AFP 0.816; combination 0.937. Validation: AKR1B10 0.840; AFP 0.789; combination 0.894 | Training early-stage vs all controls: AKR1B10 AUC 0.831, sensitivity 64.6%, specificity 92.3%; AFP AUC 0.719, sensitivity 59.5%, specificity 77.7%. Validation early-stage: AKR1B10 AUC 0.751, sensitivity 60.8%, specificity 85.7%; AFP AUC 0.595, sensitivity 32.9%, specificity 87.3% | Training AFP-negative HCC vs all controls: AKR1B10 AUC 0.891, sensitivity 71.2%, specificity 92.6%; early-stage AFP-negative HCC AUC 0.839, sensitivity 63.4%, specificity 90.7%. Validation AFP-negative HCC: AKR1B10 AUC 0.805, sensitivity 68.2%, specificity 91.0%; early-stage AFP-negative HCC AUC 0.788, sensitivity 68.0%, specificity 91.0% | Yes; independent follow-up cohort | Serum AKR1B10 outperformed AFP for HCC and early-stage HCC; combination with AFP improved diagnostic accuracy; serum AKR1B10 decreased 1 day after surgery and nearly normalized 3 days after operation | Adult evidence; supports postoperative monitoring hypothesis in pediatric HB, but no direct pediatric data |
| Du et al., 2020 (ref 14) | Quantitative proteomics; adult HCC; serum validation cohorts 1 and 2 | LC and NC | Plasma/serum AKR1B10, CTSA, AFP by ELISA | NR | NR | NR | AKR1B10 0.891; CTSA 0.894; AFP 0.831; AFP + AKR1B10 + CTSA 0.969 | NR | NR | Yes | AKR1B10 and CTSA distinguish HCC from LC/NC; combination with AFP improves AUC; serum AKR1B10 decreased after surgery | Adult evidence; no direct pediatric data |
| Wang Z et al., 2022 (ref 72) | Meta-analysis; 11 diagnostic studies, 12 cohorts; 2747 HCC, 2053 controls | Benign liver disease / controls | Serum and tissue AKR1B10; various assays | Not uniform | Overall 0.85; serum 0.80; tissue 0.78; HCC vs BLD 0.71; combined with AFP 0.84; early-stage HCC tissue 0.85 | Overall 0.78; serum 0.87; tissue 0.82; HCC vs BLD 0.84; combined with AFP 0.88; early-stage HCC tissue 0.88 | Overall 0.89; serum 0.89; tissue 0.87; HCC vs BLD 0.85; combined with AFP 0.90; early-stage HCC 0.92 | Early-stage HCC tissue: sensitivity 0.85, specificity 0.88, AUC 0.92 | NR | Yes; meta-analysis | AKR1B10 has high diagnostic value for HCC, especially early-stage HCC; combination with AFP improves accuracy | Adult evidence; no direct pediatric data |
| Matkowskyj et al., 2014 (ref 55) | Adult HCC tissues; 89 HCC, 24 hepatic adenoma, 9 focal nodular hyperplasia | Benign liver lesions (HA, FNH) | Tissue IHC; semi-quantitative 0–3+ | NR | 97% (86/89) HCC overexpressed | Benign lesions virtually absent (HA and FNH negative or minimal) | NR | NR | NR | No | Tissue AKR1B10 helps distinguish HCC from benign liver lesions; shRNA silencing increased apoptosis, decreased colony formation, enhanced doxorubicin sensitivity | Adult evidence; no direct pediatric data |
| Gao et al., 2026 (ref 9) | Case report; 2 HBV-related HCC with persistently AFP-negative | None | Serum AKR1B10; reference 0–369.30 pg/mL | NR | NR | NR | NR | Both cases had early-stage disease but were initially missed/misdiagnosed | AFP <20 ng/mL; AKR1B10 6239.29 and 1500.00 pg/mL | No; case report | Serum AKR1B10 elevated in AFP-negative HCC; dynamic changes during TACE correlated with tumor shrinkage | Adult evidence; supports combination with AFP/DCP and dynamic monitoring, but pediatric use is hypothesis |

**Supporting Table S2:** *Evidence level for proposed pediatric applications.* All proposed pediatric applications remain hypotheses. No direct pediatric evidence is currently available for any application.

| **Proposed pediatric application** | **Supporting evidence** | **Key adult studies / mechanistic basis** | **Direct pediatric evidence** |
| --- | --- | --- | --- |
| AKR1B10 may be upregulated in HB | Mechanistic inference | Adult HCC: AKR1B10 regulated by β-catenin signaling (Liu 2015, ref 17); HB: CTNNB1 mutations in 70%–90% | None |
| AKR1B10 as auxiliary serum biomarker for AFP-negative or low-expressing HB | Adult evidence | Ye 2019 (ref 13); Du 2020 (ref 14); Wang Z 2022 (ref 72); Gao 2026 (ref 9) | None |
| AKR1B10 involved in HB chemotherapy resistance | Mechanistic inference | Adult HCC and gastrointestinal cancer models: carbonyl detoxification, cisplatin/doxorubicin resistance (Matsunaga 2016, ref 68; Matkowskyj 2014, ref 55) | None |
| AKR1B10 for pHCC diagnosis | Mechanistic inference + adult evidence | Adult HCC early-stage and AFP-negative data (Ye 2019, ref 13; Wang Z 2022, ref 72); pHCC molecular similarity to adult HCC | None |
| AKR1B10 in rare pediatric liver tumors | Mechanistic inference | Adult ICC data (Heringlake 2010, ref 56; Cai 2022, ref 76); no pediatric data | None |
